# The Small Molecule Fractions of *Floccularia* *luteovirens* Induce Apoptosis of NSCLC Cells through Activating Caspase-3 Activity

**DOI:** 10.3390/ijms221910609

**Published:** 2021-09-30

**Authors:** Shuying Li, Jie Gao, Lizhen Hou, Yaxin Gao, Jing Sun, Nana Zhang, Bei Fan, Fengzhong Wang

**Affiliations:** Institute of Food Science and Technology, Chinese Academy of Agricultural Sciences, No. 2 Yuan Ming Yuan West Road, Beijing 100193, China; lishuying@caas.cn (S.L.); gj1781358244@163.com (J.G.); houlizhen2020@163.com (L.H.); gaoyx1997@163.com (Y.G.); sunjing01@caas.cn (J.S.); zhangnana@caas.cn (N.Z.); fanbei@caas.cn (B.F.)

**Keywords:** fungus, *Floccularia luteovirens*, lung cancer, NSCLC, antitumor activity, apoptosis, UFLC-Q-TOF-MS/MS

## Abstract

*Floccularia* *luteovirens* is a rare wild edible and medicinal fungus endemic to the Qinghai-Tibet Plateau. In this study, the hollow fiber membranes with molecular weights of 50 kDa, 6 kDa and 3 kDa were used to extract different fractions of *F. luteovirens*, which were named as #1, #2 and #3. Then the antitumor activity of these fractions on NSCLC cell lines, PC9 and NCI-H460, were investigated by using MTT assay, flow cytometry analysis and Western blot assay. The results indicated that the #2 and #3 fractions showed obviously inhibitory activities on PC9 and NCI-H460 tumor cells and proved that these small molecule fractions induced apoptosis of NSCLC cells by activating caspase-3. Finally, a total of 15 components, including six amino acids, two nucleosides, two glycosides, two terpenoids, one phenylpropanoid, one ester and one alkaloid, were identified in #2 and #3 fractions. This is the first evidence that the small molecule components of *F. luteovirens* were able to inhibit lung cancer by inducing apoptosis in a caspase-3 manner. The present study indicated the benefits of *F. luteovirens* in lung cancer treatment, which might be a potential resource of functional food and drugs.

## 1. Introduction

Lung cancer is the most common cancer with the highest morbidity and mortality among the human cancers worldwide [1,2]. Non-small cell lung cancer (NSCLC) is the overwhelming majority of lung cancer at 80% to 85%. Even with the recent advances in therapeutic modalities, the five-year survival rate of NSCLC patients is only 15% [3,4]. Bioactive compounds from edible fungi, also known as mushrooms, represent a new class of compounds with anticancer characters which are gaining much attention in the treatment of human cancers because of their excellent anticancer effects. This offers a new option in the development of effective preventive and therapeutic agents [5].

Edible fungi are the most popular macrofungi in the global market for their good taste, high nutritional values and some medicinal attributes [6,7]. As a healthy food, edible fungi are rich in many bioactive compounds, including polysaccharides, proteins, peptides, polyphenols, terpenoids, vitamins and dietary fiber [8,9,10], which give edible fungi a variety of functional characteristics, such as immunomodulatory, antitumor, antiviral, antibacterial, anti-parasitic, anti-oxidation and anti-diabetes properties [11,12]. Until now, humans have consumed edible fungi for more than two thousand years, and about 2000 species of edible fungi have been found in nature, but only a few species are being consumed as food, with great exploration space and potential.

*Floccularialuteovirens*, also known as “Yellow mushroom”, previously interpreted as *Armillarialuteovirens* or *Tricholoma* *luteovirens*, a well-known unique Chinese edible and medicinal macrofungus, is widely distributed in the alpine meadow in Qinghai-Tibet Plateau (QTP) where the altitude ranges from 3200 to 4800 m [13]. As an edible fungus, it is famous for its unique flavor, taste and abundant nutrients [14]. Besides these, *F. luteovirens* is frequently used for the treatment of neurasthenia, dizziness, insomnia, headaches, infantile convulsions and numbness in limbs from the traditional Tibetan medicine. *F. luteovirens* has been found to show significant biological activity against radiation, antihypoxia and antioxidants in the previous reports [15]. However, there is limited literature describing the antitumor activity of *F. luteovirens* and its components.

In this study, the different fractions were extracted from the fruiting body of *F. luteovirens* by hollow fiber membranes. Then, the antitumor activities of these fractions and their underlying mechanisms on NSCLC were investigated in vitro. Finally, the components of the effective fractions were further characterized by UFLC-Q-TOF-MS/MS.

## 2. Results

### 2.1. Extraction of Different Fractions of F. luteovirens

**Figure 1:**
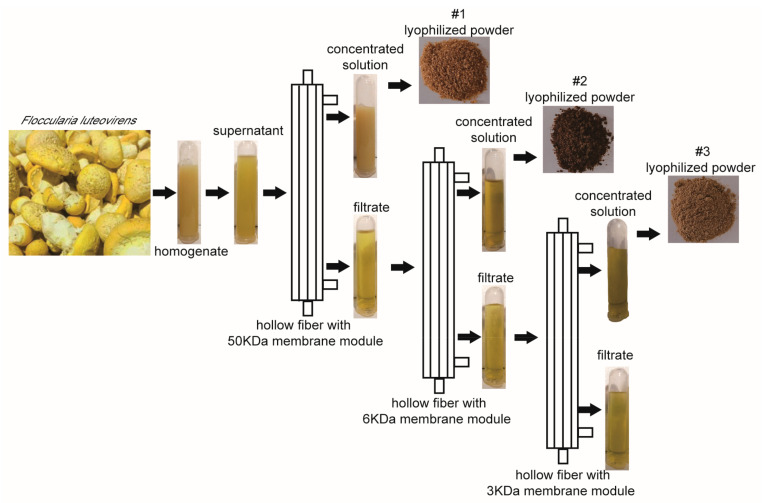
Schematic diagram of different fractions extracted from *F. luteovirens*. The obtained three fractions were labeled #1, #2 and #3, concentrated by the ultrafiltration membranes of 50 kDa, 6 kDa and 3 kDa, respectively.

The fruiting bodies of *F. luteovirens* were homogenized in 10 mM PBS buffer (pH 7.3) and extracted by hollow fiber with membrane modules of 50 kDa, 6 kDa and 3 kDa, respectively. After extraction, three fractions, recorded as #1, #2 and #3, which correspond to the concentrated solution of 50 kDa, 6 kDa and 3 kDa, respectively, were freeze-dried for subsequent analysis (Figure 1).

### 2.2. Cytotoxicity Analysis of Different Fractions of F. luteovirens on NSCLC Cells

**Figure 2:**
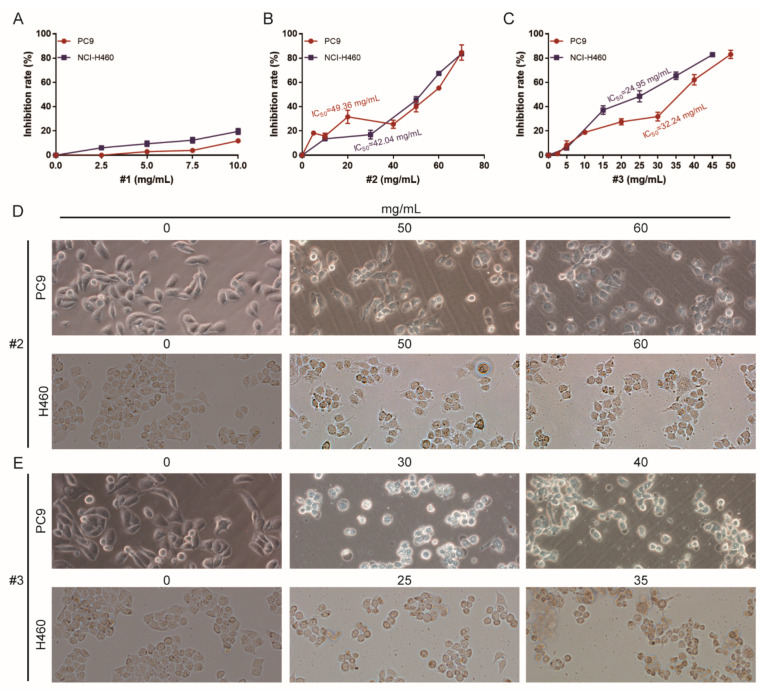
MTT analysis (**A**–**C**) and morphological observation (**D**,**E**) of different fractions of *F. luteovirens* on PC9 and NCI-H460 cells at different concentrations. The MTT results are expressed as the mean ± SD of three independent replicates.

To explore the anticancer activities of different fractions of *F. luteovirens*, cell growth was investigated with two NSCLC cell lines, PC9 and NCI-H460. Using an MTT assay, we found that three fractions of *F. luteovirens* had different cytotoxicity to these cell lines. PC9 and NCI-H460 were less sensitive to fraction #1 (Figure 2A). However, Fraction #2 and #3 were effective in inhibiting the growth of two NSCLC cell lines in a dose-dependent manner (Figure 2B,C). The IC_50_ values of #2 were 49.36 mg/mL on PC9 and 42.04 mg/mL on NCI-H460, and the IC_50_ value of #3 was 32.24 mg/mL on PC9 and 24.95 mg/mL on NCI-H460. Thus, it can be seen that NCI-H460 was more sensitive to #2 and #3 than PC9, and #3 showed more obvious cytotoxicity to the two NSCLC cell lines. Subsequently, microscopy images showed obvious cellular shrinkage after #2 and #3 treatment, with significantly decreased cellular attachment in comparison with negative control (0 mg/mL) (Figure 2D,E). These findings indicate that #2 and #3 specifically suppressed the growth of NSCLC cells and showed obvious cell type selectivity. Therefore, #2 and #3 were used for further analysis of antitumor effects and exploration of mechanisms.

### 2.3. The Apoptosis Effect of Different Fractions of F. luteovirens on NSCLC Cells

**Figure 3:**
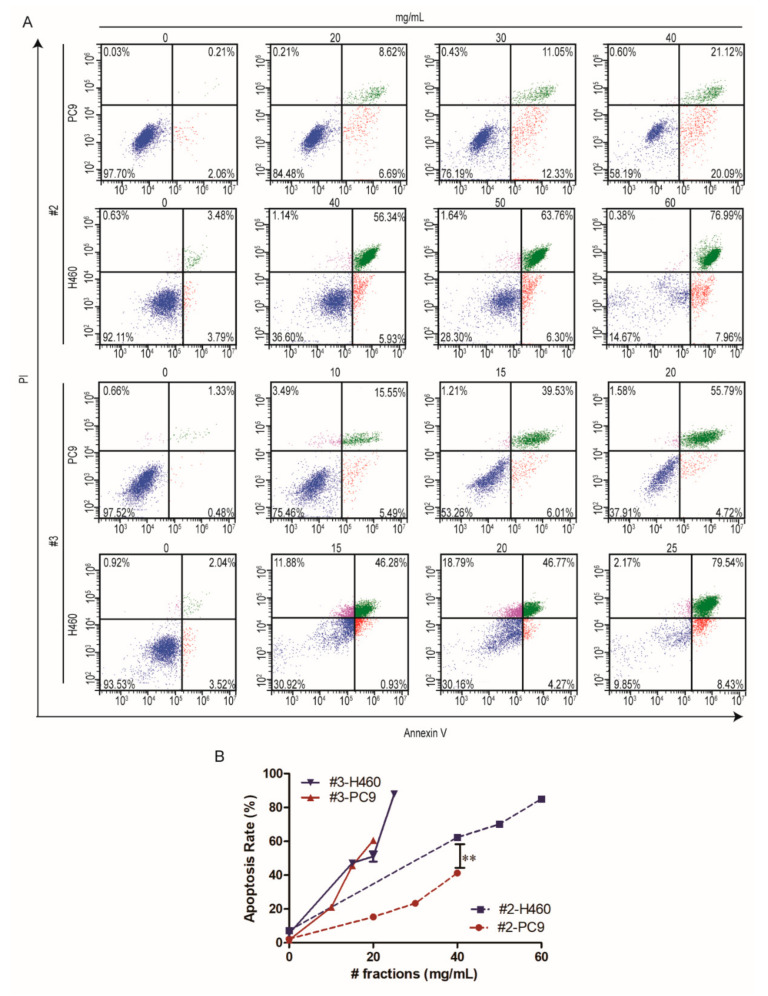
Apoptotic analysis of different fractions of *F. luteovirens* on PC9 and NCI-H460 cells at different concentrations. The induction of apoptosis was determined by flow cytometric analysis of Annexin V and PI-staining; the upper right quadrant (UR) represents late apoptotic cells stained with Annexin V and PI, and the lower right quadrant (LR) represents early apoptotic cells stained with Annexin V (**A**). The apoptosis rates were expressed as the mean ± SD of three independent replicates (**B**). **, *p*< 0.01, indicating extremely significant differences.

To confirm the apoptotic effects of #2 and #3 on NSCLC cells, we performed a flow cytometry assay after Annexin V and PI staining and the concentrations were selected by referring to the IC_50_ values. The results showed that #2 and #3 caused both early and late apoptosis in a dose-dependent manner. As shown in Figure 3, the NCI-H460 cells showed obviously apoptotic effects upon #2 and #3 treatments compared to those of PC9, and the #3 treatment caused more remarkable apoptotic effects to both NSCLC cell lines, which was consistent with the results of the cytotoxicity assay. Taken together, these results indicated that #2 and #3 could induce apoptosis in NSCLC cell lines.

### 2.4. The Apoptosis Mechanism of Different Fractions of F. luteovirens on NSCLC Cells

**Figure 4:**
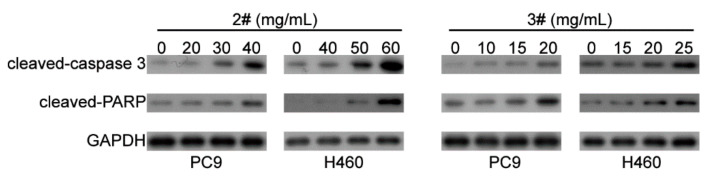
Western blot analysis of apoptosis-related proteins of PC9 and NCI-H460 cells treated with different fractions of *F. luteovirens* at different concentrations.

In order to further clarify the mechanism of the apoptosis effects of #2 and #3 fractions of *F. luteovirens*, the expression levels of apoptosis-related proteins caspase-3 and PARP were detected by Western blot. The results showed that the degradation products of caspase-3 (cleaved-Caspase3) and PARP (cleaved-PARP) were significantly upregulated with the increased dose of #2 and #3 (Figure 4). This suggested that #2 and #3 induced caspase-mediated apoptosis in NSCLC cells.

### 2.5. Chemical Profiling of Different Fractions of F. luteovirens

**Figure 5:**
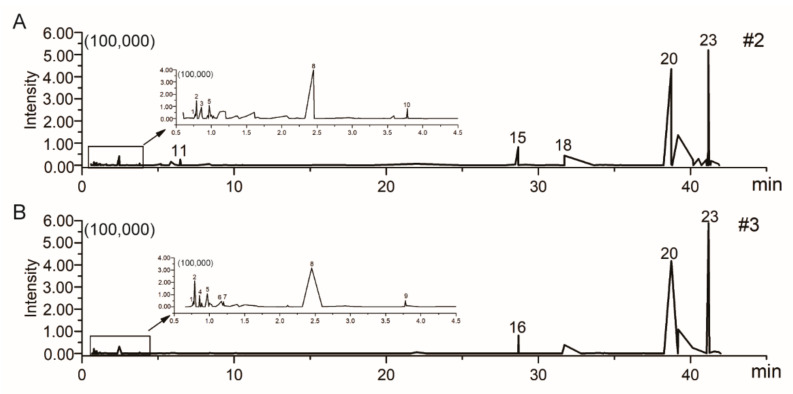
The total ion chromatogram (TIC) of UFLC-Q-TOF-MS/MS obtained from #2 (**A**) and #3 (**B**) in the positive-ion mode.

The chemical constituents of #2 and #3 were determined by UFLC-Q-TOF-MS/MS to further analyze their potential antitumor components. The positive ion modes of #2 and #3 were collected, and the ion flow diagram of the full scan is shown in Figure 5. The obtained results were imported into the UNIFI software and the compounds met the mass error of less than 10 ppm with correct isotope distribution, and the second-order fragments were identified as target substances [16,17,18]. The compound description, formula, elemental composition and characteristic fragment ions of #2 and #3 are shown in Table 1. In the end, a total of 15 compounds were identified in #2 and #3 fractions, including six amino acids, two nucleosides, two glycosides, two terpenoids, one phenylpropanoid, one ester and one alkaloid. Among them, glycosides were detected only in #2, phenylpropanoid and ester were detected only in #3, and all the others were detected both in #2 and #3.

## 3. Discussion

This is the first report of the extraction of three fractions, named #1, #2 and #3, from the fruit body of *F. luteovirens* by hollow fiber membrane. This study proved that the small molecule fractions of #2 and #3 had stronger antitumor activity and cell selectivity on two types of NSCLC cell lines in a dose-dependence manner. Further exploration of the antitumor effect and mechanism confirmed that these two small molecular fractions induced apoptosis of NSCLC cells by activating caspase-3. Finally, a total of 15 compounds were identified in #2 and #3 fractions.

Green and sustainable preparation of membrane separation technology will be more competitive in the context of advocating green manufacturing, because higher large-scale mechanized operation properties can be achieved by using this approach; moreover, it has a high separation efficiency, low energy consumption, is environmentally friendly and is an economical process [19]. In addition, use of hydrophilic solutions such as PBS to enrich and extract functional factors will neither destroy the separated components nor bring in harmful components. The high-speed development of advanced membrane separation technology makes it more and more applied to the research and development of functional food. In the present study, this technique proved to be efficient for extraction of fractions with different molecular weights from the fruit body of *F. luteovirens*.

*F. luteovirens* is a rare wild resource, usually consumed and sold by fresh or dried types. As an edible and medicinal fungus, *F. luteovirens* is rich in nutrients and shows certain therapeutic potential for variety of diseases. The extraction and identification of more functional ingredients will contribute to the development of higher value-added functional foods and pharmaceuticals. Wild edible macro fungi *F. luteovirens* have been proved to be a valuable source for the identification of novel lead molecules with therapeutic potential. Recent studies indicated that macromolecule lectin and polysaccharide of *F. luteovirens* possess antitumor and antioxidant activities [15,20]. In this study, we first proved that the small molecule fractions of #2 and #3 from *F. luteovirens* had obvious cytotoxicity on NSCLC cell lines. The findings indicated that the fruit body of *F. luteovirens* could be used as a potential natural source of anti-cancer agents.

In-depth exploration of the antitumor effect and mechanism is a prerequisite of industrial development. Apoptosis is a programmed cell death which is also regarded as the preferred way to eliminate cancer cells [21]. Caspase-3 is a critical molecule for stimulating apoptosis of cancer. Pro-caspase 3 is cleaved to form active caspase-3, the cleaved caspase-3, which is the main cleavage enzyme to promote apoptosis [22,23,24]. PARP, the critical substrate of caspase-3, is involved in repair and gene integrity monitoring. At the initiation of apoptosis, the PARP is cut into two fragments by caspase-3, leading to the two zinc finger structures bound to DNA in PARP separated from the catalytic region of the shuttle end and not being able to perform normal functions [25]. As a result, the activity of the Ca^2+^/Mg^2+^ dependent endonuclease affected by the negative regulation is increased, and the DNA between the nucleosomes is cleaved, eventually causing apoptosis [26]. In this study, the flow cytometry analysis clarified that the small molecule fractions of #2 and #3 from *F. luteovirens* caused NSCLC cells death by inducing apoptosis, and the Western blot assay further elucidated that they induced NSCLC cell apoptosis by activating the caspase-mediated manner.

Aiming to clarify the antitumor constituents of *F. luteovirens*, UFLC-Q-TOF-MS/MS was used to analyze the fractions of #2 and #3. A total of 15 compounds, including six amino acids, two nucleosides, two glycosides, two terpenoids, one phenylpropanoid, one ester and one alkaloid, were identified in #2 and #3 fractions. However, the exact chemical constituent(s) responsible for the observed activity are currently unexplored. The complex chemical composition of *F. luteovirens* makes the isolation of bioactive constituents very difficult, so this kind of information is scarcely reported. Therefore, the methods for isolation of individual compounds from *F. luteovirens* are required for further studies.

## 4. Materials and Methods

### 4.1. Extraction of Different Fractions of F. luteovirens

The fruiting bodies of *F. luteovirens* used in this study were obtained from Tibet Academy of Agricultural and Animal Husbandry Sciences. The fresh fruiting bodies of *F. luteovirens* were homogenized in 10 mM PBS buffer (pH7.3) with a solid: liquid ratio of 1:3 and extracted at 4 °C overnight. Then the extracting solution was centrifuged at 4625× *g* for 10 min. The supernatant of *F. luteovirens* was filtered by hollow fiber with a membrane module of 50 kDa, 6 kDa and 3 kDa, respectively. The ultrafiltration pressure was controlled at 0.1 MPa and the temperature was 4 °C. After ultrafiltration, three fractions were freeze-dried for subsequent analysis.

### 4.2. Tumor Cell Culture

Human NSCLC cell lines with EGFR mutations (PC9) or with wild-type EGFR (NCI-H460) were used in this study [27]. The purpose was to determine whether the three fractions of *F. luteovirens* had an inhibitory effect on both EGFR-mutant NSCLC and EGFR wild-type NSCLC. PC9 and NCI-H460 cells were obtained from the American Type Culture Collection (ATCC, Manassas, VA, USA). The cells were cultured in DMEM (Gibco, Rockville, MD, USA), supplemented with 10% fetal bovine serum (FBS; Life Technologies, Inc., Rockville, MD, USA), 100 μg/mL streptomycin, and 100 U/mL penicillin (Life Technologies, Inc., Rockville, MD, USA) at 37 °C in a humidified incubator containing 5% CO_2_. Cells were sub-cultured at 80–90% culture solution and were used for experiments in the exponential growth phase.

### 4.3. Sample Preparation for the Anti-Lung Cancer Detections

The three fractions showed different solubility due to the different molecular weights. The solubility of #1 was the worst, while #2 and #3 showed better solubility. In the following anti-lung cancer experiments, the freeze-dried samples of three fractions of *F. luteovirens* were dissolved in 10 mM PBS (pH7.3) buffer by maximum concentration and then double diluted. Therefore, different concentrations of the three fractions were selected for different experiments. PBS buffer was used as the negative control.

### 4.4. Tumor Cell Proliferative Inhibitory Assay

The PC9 and NCI-H460 cells were seeded into 96-well plates in 1 × 10^5^ cells/well, incubated for 24 h and then exposed to the different fractions of *F. luteovirens* or negative control for 24 h. Cell viabilities were evaluated with an MTT assay (Sigma, St. Louis, MO, USA). In brief, 10 μL of 5 mg/mL MTT was added to each well and incubated for 4 h. The medium was replaced with 150 μL of DMSO to dissolve the crystal formazan dye, and absorbance was detected at 490 nm using an ELX808IU Microplate Reader (Bio-Tek, Winooski, VT, USA). The inhibition rates were calculated as follows: inhibition rate (%) = (average A_490_ in the control group−average A_490_ in the experimental group)/(average A_490_ in the control group−average A_490_ in the blank group) × 100%. The IC_50_ value was determined as the concentration that caused 50% inhibition of cell proliferation.

### 4.5. Tumor Cell Morphological Observation

For observing the morphology, cells were seeded into 12-well plates in 1 × 10^6^ cells/well, cultured for 24 h and then exposed to the three fractions of *F. luteovirens* with different concentrations for 24 h. The morphological changes of the tumor cells were observed under light microscope (IX70; Olympus Corporation, Tokyo, Japan) and pictures were taken with a digital camera at 20× lens.

### 4.6. Tumor Cell Apoptosis Assay

PC9 and NCI-H460 cells (4 × 10^5^ cells/well) were seeded into a 6-well plate and cultured for 24 h. Then three fractions with different concentrations were added. After culturing for another 24 h, the apoptosis rate (percentage of apoptotic cells) was evaluated using the Annexin V-FITC/PI Cell Apoptosis Detection Kit (BD Biosciences, San Jose, CA, USA) with flow cytometry. Staining was conducted in accordance with the manufacturer’s protocol. The stained cells were then analyzed using a flow cytometer (Becton Dickinson, San Jose, CA, USA) with Cellquest software.

### 4.7. Western Blot Analysis

To investigate the potential apoptotic induction mechanism, the expression of the specific apoptosis related proteins was measured using the Western blot test [28]. The treated tumor cells were lysed with ice-cold RIPA buffer (Thermo Fisher Scientific, Hannover Park, IL, USA). The supernatants were collected after cell lysates by centrifugation at 13,523× *g* for 10 min at 4 °C, and the protein concentration were measured by a BCA method. Then the corresponding volume of SDS was added and boiled at 99 °C for 5 min to make the protein denatured. After SDS-PAGE electrophoresis, the PVDF membrane was transferred, sealed with 5% skim milk, the primary antibody was incubated at 4 °C overnight and the secondary antibody labeled with horseradish peroxidase was incubated at room temperature for 2 h. Protein signals were detected using ECL Western Blotting Detection Kit (Minneapolis, MN, USA). Primary antibodies against PARP, cleaved caspase-3, GAPDH and the secondary antibody (goat anti-rabbit antibody conjugated to horseradish peroxidase) were all purchased from Cell Signaling Technology, Inc. (Beverly, MA, USA).

### 4.8. UFLC-Q-TOF-MS/MS Analysis

Sample preparation referenced the reported literature [29], with slight modifications as follows. An appropriate number of samples were accurately weighed and dissolved in methanol with a concentration of 2 mg/mL. The samples solution was then treated by ultrasonication for 30 min. The precipitate was removed by centrifugation at 13,523× *g* for 15 min. The supernatant samples were filtered by a 0.22 μm microporous filter membrane.

The UFLC analysis was performed on an ACQUITY UPLC system (Waters, Milford, MA, USA) equipped with an integral column heater and a binary solvent pump. An ACQUITY UPLC HSS T3 column (2.1 mm ×100 mm, 1.8 μm) was applied for all analyses with oven temperature at 35 °C. The flow rate was 0.3 mL/min, which consisted of solvent A (0.1% formic acid in water) and mobile phase B (acetonitrile). The mobile phase gradient was as follows: 5% B-95% A for 35 min, 95% B-100% B for 1 min, 100% B for 4 min, 100% B-5% B for 1 min, 5% B for 9.0 min. The injection volume of each sample solution was 3 μL.

A Waters Xevo G2 Q-TOF mass spectrometer (Waters, Milford, MA, USA) coupled with an ESI source was used for the MS/MS analysis. Data was acquired and analyzed with Waters Mass Lynx V4.1 software. The ionization source conditions were as follows: the capillary voltage and sampling cone were set at 3 kV and 40 V. The source and desolventing temperatures were set at 100 and 500 °C, respectively. The flow rates of cone gas (N_2_) and desolvation gas (N_2_) were 100 and 950 L/h, respectively. The MS data were collected from m/z 50–1200 mDa in positive ionization mode. The acquisition time was 0–60 min and the scanning time was 0.1 s.

### 4.9. Statistical Analysis

All treatments were performed in triplicates, and data are expressed as mean ± SD. Mean and SD were calculated by Microsoft Excel 2016 software (Microsoft, Redmond, WA, USA). Statistical comparisons were made by a two-tailed Student’s t-test using IBM SPSS 13 software (Version 22, New York, NY, USA). Differences were regarded as statistically significant for *p* < 0.05 and extremely relevant for *p* < 0.01. The line graphs were generated by GraphPad Prism 5 software (GraphPad Software Inc., La Jolla, CA, USA). The combined graphs were generated by Adobe Photoshop CS5 and Adobe Illustrator CS5 (Adobe Systems Inc., San Jose, CA, USA).

## 5. Conclusions

In this study, three fractions of *F. luteovirens* with different molecular weights were extracted by membrane separation technology. The cytotoxicity evaluation proved that the small molecule fractions of #2 and #3 from *F. luteovirens* showed robust antitumor activity on NSCLC cell lines, PC9 and NCI-H460. Further research on the antitumor effects and mechanisms demonstrated that these small molecule fractions stimulated apoptosis of NSCLC cells through activating caspase-3. Finally, a total of 15 compounds were identified in #2 and #3 fractions. This is the first evidence that the small molecule fractions of *F. luteovirens* are able to inhibit lung cancer by inducing apoptosis in a caspase-3 manner. In summary, *F. luteovirens* may be a potential natural resource for lung cancer therapy.

## Figures and Tables

**Table 1:** The different components of #2 and #3 fractions from *F. luteovirens* analyzed by UFLC-Q-TOF-MS/MS in positive mode.

No.	[M + H]^+^(*m*/*z*)			Proposed Formula	Identification	Fragment Ions (MS^2^) *m*/*z*	Source	Type
	Measured	Predicted	Error (ppm)					
1	156.0771	156.0770	0.64	C_6_H_9_N_3_O_2_	Histidine	110.0706	#2, #3	amino acid
2	175.1186	175.1184	1.14	C_6_H_14_N_4_O_2_	Arginine	116.0706,129.1030	#2, #3	
3	148.0613	148.0614	−0.68	C_5_H_9_NO_4_	Glutamic acid	130.0509,114.0555	#2	
4	182.0813	182.0810	1.64	C_9_H_11_NO_3_	Tyrosine	91.0542	#3	
5	166.0866	166.0863	1.8	C_9_H_11_NO_2_	Phenylalanine	120.0803	#2, #3	
6	205.0969	205.0972	0.9	C_11_H_12_N_2_O_2_	Tryptophane	188.0695	#2	
7	267.1387	267.1379	2.9	C_18_H_18_O_2_	Honokiol	115.0540	#3	phenylpropanoid
8	268.1041	268.1040	0.1	C_5_H_5_N_5_	Adenine	136.0599	#2, #3	nucleoside
9	269.0870	269.0881	3.9	C_5_H_4_N_4_O	Hypoxanthine	137.0444	#3	
10	279.1614	279.1617	−1.07	C_16_H_22_O_4_	Dibutyl phthalate	149.0230	#3	ester
11	315.1074	315.1085	3.61	C_14_H_20_O_8_	Cimidahurinine	149.0233	#2	glycoside
12	827.4469	827.4424	5.4	C_42_H_66_O_16_	Esculentoside A	425.2153	#2	
13	336.3289	336.3294	−1.5	C_22_H_41_NO	*N*-Isobutyl-(2E,4E)- octadecadienamide	135.1173	#2, #3	alkaloid
14	339.2540	339.2530	2.8	C_20_H_34_O_4_	Kirenol	221.2268	#2, #3	terpenoid
15	437.2353	437.2322	6.9	C_27_H_32_O_5_	5-*O*-Benzoyl-20- deoxyingenol	133.0851	#2	

## Data Availability

Not applicable.
